# Remarkable Case of Tumor of the Side

**Published:** 1856-01

**Authors:** E. J. Roach

**Affiliations:** Atlanta, Ga.


*Remarkable case of Tumor of the Side.* By E. J. Roach, M. D., Atlanta, Ga.

Sometime in June last, I was consulted by the Rev. Mr. ——, aged fifty-six, concerning a tumor located in the right hypochondric region. Upon examination, I discovered it to be a hard, immovable substance, about six inches in circumference, very sensible to manipulation.

The gentleman stated that it first began to make its appearance about a year previous to the above mentioned date; but for six months prior to this time, he was troubled occasionally with pain in this region, which continued to embarrass him up to the period of my examination, sometimes almost amounting to spasms.

Supposing the case to be a very interesting one, and not being able at the time to form a correct diagnosis, but thinking probable that it had some connection with the liver, paliative treatment was recommended, and the patient discharged.—About three weeks after the above interview, I was called, late at night, to the patient. On my arrival, I found him in spasms, suffering intense pain. The muscles in the immediate region of the tumor were violently contracted, so much so that he was in the act of vomiting; this being assisted by the aid of an emetic, he received temporary relief. After ordering warm fomentations to the side and administering an anodine I left.

The day following, I learned that he had rested well since my last visit. The tumor presented much of the same appearance as it did on my first seeing him. He was now ordered the use of Potassæ Iodidi internally, and Lugal's Solution externally. This treatment he continued for several days without relief. During this time the symptoms became more aggravated with more frequent return of the spasms. The tumor still continued hard, and the patient very emaciated, frequent, with scarcely perceptable pulse. I now thought it judicious to open it, and called my friend Dr. D—— in consultation, who concurred in my opinion. The tumor was accordingly opened, and discharged, I think, a pint of pus within fifteen minutes, having none of the characteristics of bile, save its color. The patient was again ordered the use of the poultice.

The next day, on visiting the patient, I found him much better; I suppose during the interval of opening it until the next morning, about twelve hours, it discharged nearly a half gallon. I now proceeded to explore it carefully, but, to my great surpise, could find no internal connection whatever, even with the most delicate probe. I then dilated the orifice large enough to admit my finger, still my effort was fruitless to find a connection. A tent was inserted, and the patient continued to improve. He has been well ever since, bating an attack of intermittent fever, and an occasional muscular contraction of the side. He continues to wear the tent.

